# A Host-Directed Approach to the Detection of Infection in Hard-to-Heal Wounds

**DOI:** 10.3390/diagnostics12102408

**Published:** 2022-10-04

**Authors:** Michael Burnet, Daniel G. Metcalf, Scarlet Milo, Clemens Gamerith, Andrea Heinzle, Eva Sigl, Kornelia Eitel, Marieke Haalboom, Philip G. Bowler

**Affiliations:** 1 Synovo GmbH, Paul Ehrlich Straße 15, 72076 Tuebingen, Germany; 2 ConvaTec Ltd., First Avenue, Deeside Industrial Park, Deeside CH5 2NU, UK; 3 Austrian Centre of Industrial Biotechnology, Krennagsse 37, A-8010 Graz, Austria; 4 Qualizyme Diagnostics GmbH & Co. KG, Neue Stiftingtalstrasse 2, A-8010 Graz, Austria; 5 Medical School Twente, Medisch Spectrum Twente, 7512 KZ Enschede, The Netherlands; 6 Phil Bowler Consulting Ltd., Warrington WA4 5QZ, UK

**Keywords:** wound, infection, hard-to-heal, chronic, inflammation, neutrophil, myeloperoxidase, lysozyme, elastase, cathepsin G

## Abstract

Wound infection is traditionally defined primarily by visual clinical signs, and secondarily by microbiological analysis of wound samples. However, these approaches have serious limitations in determining wound infection status, particularly in early phases or complex, chronic, hard-to-heal wounds. Early or predictive patient-derived biomarkers of wound infection would enable more timely and appropriate intervention. The observation that immune activation is one of the earliest responses to pathogen activity suggests that immune markers may indicate wound infection earlier and more reliably than by investigating potential pathogens themselves. One of the earliest immune responses is that of the innate immune cells (neutrophils) that are recruited to sites of infection by signals associated with cell damage. During acute infection, the neutrophils produce oxygen radicals and enzymes that either directly or indirectly destroy invading pathogens. These granular enzymes vary with cell type but include elastase, myeloperoxidase, lysozyme, and cathepsin G. Various clinical studies have demonstrated that collectively, these enzymes, are sensitive and reliable markers of both early-onset phases and established infections. The detection of innate immune cell enzymes in hard-to-heal wounds at point of care offers a new, simple, and effective approach to determining wound infection status and may offer significant advantages over uncertainties associated with clinical judgement, and the questionable value of wound microbiology. Additionally, by facilitating the detection of early wound infection, prompt, local wound hygiene interventions will likely enhance infection resolution and wound healing, reduce the requirement for systemic antibiotic therapy, and support antimicrobial stewardship initiatives in wound care.

## 1. Introduction

Invasion of wounded dermal tissue by a pathogen, or group of pathogens, induces a host inflammatory response which manifests as acute clinical signs such as pain, heat, redness, and swelling [1]. In this situation, acute inflammation is the patient’s natural protective response to eliminate the causative pathogen(s) and initiate wound healing. When the inflammatory response is unable to suppress an infection, empirical antibiotic therapy is typically administered. Additionally, microbiological analysis of associated tissue or fluid is often undertaken subsequently to confirm pathogen identity and justify appropriate antibiotic therapy [2].

Although the clinical signs of acute infection are invariably evident on visual inspection, recognizing signs of infection in chronic or hard-to-heal wounds is much more challenging and has been the subject of debate since the 1990s [3]. Although inflammation is a natural response to pathogens in both acute and chronic wounds, their clinical manifestations are very different, i.e., visually obvious in acute wounds, but considerably less so in chronic wounds [4,5]. Consequently, determining infection status in hard-to-heal wounds is difficult, and considerable variation exists amongst health care providers (HCPs) in clinical diagnosis [6].

Neither causative pathogens nor their antimicrobial susceptibility profile can be anticipated based on the clinical presentation of a wound infection. This uncertainty will often prompt HCPs to take a swab for microbiological investigation, but given the diversity and complexity of hard-to-heal wound microflora [2], it is not usually possible to identify the causative pathogen(s) [7]. Relying on traditional microbiological culture will often lead to inaccuracies in infection diagnosis, and hence inappropriate treatment, or necessary treatment being withheld [8]. Such treatment pathways are a barrier to optimal antimicrobial use, since the uncertainty associated with nonspecific presentations often leads to the widespread and indiscriminate use of antibiotics. 

Determining infection status in complex and hard-to-heal wounds via clinical judgement and/or microbiological investigation are long-standing practices that are often subjective, variable, and inaccurate. Consequently, it is necessary to identify new clinical approaches that can assist HCPs to determine wound infection status more confidently and therefore guide optimal treatment. 

Rapid diagnostic tests based on highly conserved biomarkers of wound infection may provide less ambiguous indicators of a pathological process compared with visual inspection or polymicrobial microbial culture. Their use could reduce uncertainty and assist clinicians in practicing improved antibiotic stewardship and judicious use of antiseptic dressings. A collaborative partnership between diagnostic stewardship at the point-of-care and antimicrobial safeguarding will allow appropriate adjustment of antimicrobial regimens that allow diagnostic test results to translate into improved patient care.

Since inflammation is a natural response to pathogen interference in wounds, it appears relevant to more closely investigate the relationship between microbial activity and host inflammatory response in the wound environment. To date, research in this field is encouraging [9,10,11,12,13,14]. The aim of this review is to address the mechanisms of wound infection diagnosis in more detail and introduce the clinical application of host inflammatory markers to detect infection status, particularly in complex and hard-to-heal wounds.

## 2. Wound Infection

Wound infection is a host inflammatory response to microbial interference [5]; it is not necessarily a consequence of the presence or abundance of micro-organisms within a wound [7]. Microbial interference and resulting inflammation may manifest directly or indirectly within a wound environment [5]. Actively metabolizing and invading planktonic bacteria provoke a direct host inflammatory response (neutrophil infiltration) that manifests as obvious signs of inflammation, namely heat, redness, pain, and swelling [5]. In contrast, bacteria existing in biofilm form, which is a primary cause of, and hence prevalent in, chronic, hard-to-heal wounds, induce a less visually obvious but persistent inflammatory response [5].

Acute infection as might occur following trauma or surgery is characterized by rapid multiplication and invasion of planktonic organisms in viable tissue where relatively rapid onset and fulminant development makes traditional visible diagnosis obvious within a few hours to days [2]. In contrast, macroscopic signs and symptoms of infection in hard-to-heal wounds may evolve slowly, and often the signs remain subtle and ambiguous as the wound progresses to a persistent, hyper-inflammatory state [1]. Whilst physiological co-morbidities were long considered to be the main cause of chronic status, there is a trend to revisit the idea that chronic infection and biofilm involvement may be playing a greater role in delaying healing [15]. In slow-developing infections with masked or ambiguous signs, the inability to detect and therefore treat the infection early leads to a potential lost opportunity to manage these infections more efficiently and effectively. 

We propose that detecting early/incipient infection would allow timely local wound hygiene and non-antibiotic-based therapy that would both simplify and improve wound treatment outcomes in a cost-effective manner. Fluctuations in the individual patient’s levels of immune and host defense biomarkers should be reflected in the techniques used to rapidly detect infection at the point of care.

### 2.1. Distinguishing Colonization from Infection

Colonization is a term that indicates the presence of an organism without necessarily interfering with wound healing [16]. When or whether colonization leads to infection is often not clear and appears to be dependent on both microbiological and host factors [17]. However, apart from clinical signs, there are few tools for observation and prediction of the transition to infection in a timely manner.

The term “critical colonization” has attracted significant attention and scrutiny in recent years and is often dismissed or regarded as synonymous with local infection [16,18,19]. Nevertheless, the scientific underpinning of the concept of critical colonization lies in the delay of wound healing by microbial factors (e.g., biofilm, or toxins that reduce innate immune response) without the overt and clinically obvious signs of infection. Failure to identify the shift from wound colonization to wound infection (pathogen invasion) impedes timely diagnosis, thus delaying appropriate treatment and wound healing.

### 2.2. The Role of Biofilm in Chronic Infection

The term biofilm is widely used to describe surface-associated microbial communities, comprising various organisms and growth forms within a three-dimensional matrix of extracellular polymeric substances (EPS). EPS provides the organisms with protection from external threats such as cellular and chemical antimicrobial agents. Biofilm plays a significant role in the inability of chronic, hard-to-heal wounds to progress towards healing [20,21,22]. Alteration of gene expression and gene products within biofilm are responsible for persistent inflammation [23], antibiotic tolerance [22,24,25] and evasion of host adaptive and innate immunity [26]. However, no individual gene or technology can be used to identify the biofilm mode of growth. Furthermore, persister cells arise within biofilm, owing to a state of metabolic dormancy [27,28]. Persister cells in biofilm appear to contribute to the recalcitrance of chronic infections in that their metabolic quiescence protects them from antimicrobial substances but allows resumption of activity once antimicrobials have been discontinued [29]. 

One of the most significant barriers to effective biofilm management lies in the most commonly used diagnostic tool for wound infection, namely culture and viable counts. This is ill-suited to the detection of biofilm due to issues of sampling, separation, mutualism, and metabolic dormancy, rendering biofilm cells difficult to culture via traditional methods [30]. This has caused a paradigm shift within clinical wound management to account for the presence of biofilm, although in many cases this manifests as an observational “trial and error” approach [21]. While the link between pathogenic microorganisms and infection has been understood for over a century, the link between wound infection and biofilm has only recently been understood [6,31,32,33]. 

Over the last decade, greater emphasis has been placed on the role of multidrug-resistant organisms and biofilms. These cause over 90% of chronic wound infections [30,34]. In addition to the other factors described above, the impact of biofilm can be exacerbated by horizontally inherited antibiotic resistance traits. These include membrane-associated efflux pumps (which prevent the accumulation of lethal concentrations of antimicrobial agents), alternation of target proteins, methylation of ribosomes, and antibiotic degradation enzymes, such as beta-lactamase that degrade beta-lactam antibiotics [35,36]. 

### 2.3. Current Approaches in the Diagnosis of Wound Infection Status

In addition to the dual challenge of antibiotic resistance and biofilm [25], infection is a central concern amongst wound care providers owing to the patient burden, treatment costs and demand on care resources for its management. While prevention of infection has always been central, there is a growing emphasis on improving management strategies in a two-pronged approach. Firstly, improved wound infection treatment, and secondly, improved detection and damage limitation, or prevention via earlier therapeutic initiation. This has driven demand for accurate wound infection diagnosis, to provide meaningful data and subsequent treatment pathways to caregivers [37,38]. This has been partly frustrated by the fact that wound infection is typically polymicrobial [39] and often driven by biofilm. Therefore, alongside extant methods of clinical and microbiological assessment, new approaches have been examined in more detail.

#### 2.3.1. Clinical Observation

Visible signs of infection-induced inflammation are familiar and can be directly related to underlying immune processes. Acute infection occurs when virulence factors in one or more microorganisms neutralize or evade the patient’s innate and adaptive immune systems. Subsequently, invasion and dissemination of metabolically active (planktonic) microorganisms in viable tissue provoke a series of local and systemic host responses that manifest as heat, pain, edema and erythema [2,40]. The qualitative diagnosis of wound infection frequently involves the identification of such clinical signs; hence its early detection relies heavily on the skill and experience of the HCP. 

The key conceptual issue that emerges is that wound infection is still broadly considered to be a state in which the wound is visibly infected, namely, that inflammation, suppuration, and pain are so advanced as to be obvious to patient and HCP alike [2]. In reality, and particularly in chronic wound infections, the total time period of a wound infection is likely to be longer than is visibly recognized because clinical signs and symptoms take time to become apparent. Thus, what we currently consider to be “infection” may be more accurately considered a severe or established infection. While acute wound infections tend to develop more rapidly with obvious signs of infection-induced inflammation, chronic wound infections manifest very differently [5]. Because biofilm is the root of the problem, as a foreign body it induces the infiltration of neutrophils as occurs in acute wounds. However, since biofilm matrix protects associated bacteria, neutrophils accumulate around the biofilm, becoming “frustrated” in their inability to thwart microbial onslaught. Neutrophil activity around the biofilm results in the release of antimicrobial oxygen metabolites and enzymes, that ultimately destroys host tissue, and providing an additional nutrient source for the evolving biofilm [5]. Wound biofilm thus enjoys a parasitic relationship with the host, taking control of host inflammation and using it to its benefit [5]. Biofilm-induced chronic wounds consequently manifest as a persistent hyper-inflammatory condition, with subtle clinical signs including sullen/dark granulation tissue, friable granulation tissue, malodor, and delayed healing [5]. Any delay in diagnosis is partly because these signs are subjective, and often require examination of the wound and patient over a prolonged period to observe changes.

Despite the subjectivity, most practitioners rely on clinical signs and symptoms to diagnose wound infection (98% of the time), followed by patient-reported symptoms (88% of the time) [41], yet these HCPs still commonly use wound cultures in an attempt to confirm infection status [38].

#### 2.3.2. Microbiological Investigation

The current approach to confirming infection by enumerating and/or identifying organisms is based on the concept that infection is associated with the abundance of microorganisms or the presence of specific pathogens. However, enumeration does not correlate with infection status [7] and does not address the relative pathogenicity of isolates [42], nor the position of organisms in the wound profile (potentially confounding opportunists at the surface interface with potential pathogens in the wound bed) [43,44]. Punch biopsy partly mitigates these issues, but it is too invasive and time-consuming for routine monitoring and has the potential to spread infection and cause pain [2]. There is also evidence that punch biopsy and surface swab are similar in terms of recovery/organism specification [6]. 

Whilst culture is often justified with the argument that knowing the causative organism will aid in the selection of an appropriate treatment pathway, this is less relevant in general wound therapy practice where first- and second-line approaches are usually pre-defined [45], and most infections are initially polymicrobial [32]. In addition, causative organisms are often anaerobic bacteria that are notoriously difficult to culture in vitro, thus often overlooked despite their significant contribution to microbial biomass and pathogenicity [46]. Enumeration also under-represents other unculturable or hard-to-culture organisms [47] which is often associated with biofilm [30]. 

Consequently, microbial culture is only weakly predictive in practice, providing results that are easily interpreted only when the infection is already clinically obvious (and where a significant over-growth of one organism is apparent) [2,18]. Therefore, culture is often, at best, only weakly confirmatory and rarely yields a clear causation-treatment nexus. Indeed, given that it can often take several days to obtain results from microbiology culture, most first-line antibiotic therapy is applied before any microbiology results are available. 

In early, local, polymicrobial infections without a dominant pathogen, the application of broad-spectrum antimicrobials is combined with a “wait-and-see” approach. Since antibiotic therapy often selects for the emergence of a dominant pathogen, one value of microbiological culture then lies in the evaluation of antibiotic susceptibility profiles, providing useful information to prescribe the most efficient antibiotic treatment [48].

#### 2.3.3. PCR and Sequencing-Based Technologies

Acute bacterial infections in general medicine often involve single species [2,49]; thus, identification of the causative organism can be helpful in selecting therapy, as it is likely that similar symptoms are associated with the same pathogen in a given area and time. Taking community-acquired pneumonia as an example, the identification of causative organisms can trigger the use of defined treatment protocols [50]. However, chronic wound infection differs in that colonizing microorganisms originate from a variety of sources including surrounding skin, mouth, gut, and the environment, and consequently, this rarely leads to a single pathogen dominating the infection [7]. 

Given this complexity, total sample DNA sequencing is a potentially unbiased means to enumerate and classify a microbial community. It has been widely applied at the research level in studies of the gut [51], and initial data sets from wound infection sites have been obtained [52,53]. These data show significant diversity but provide indications that certain classes of organisms such as enterobacteria and facultative anaerobes in general are associated with non-healing wounds [54]. Polymerase chain reaction (PCR)-based diagnostics and mixed primer panels (e.g., for 16S variants) have the potential to identify and quantify organisms present with good sensitivity [47,55,56]. In ideal cases, PCR can also detect known resistance genes of common organisms [52]. These detailed outcomes are based on a degree of supposition of likely pathogens and strains. Whilst it would be theoretically possible to create diagnostic primers for most organisms and strains typically found in wounds, it remains more practical to use defined primer panels for hypothesis-driven identification of pathogens present. Sequencing and PCR avoid the bias against anaerobes and fastidious organisms observed in culturing techniques and provide more realistic indications of microbial diversity and abundance. Nonetheless, they are resource intensive, and their use is still only justified once a clear case for infection exists. Establishing this case in complex hard-to-heal wounds remains the key problem to solve.

The advantages of these techniques are unfortunately outweighed by several disadvantages. These systems require clean samples and can be affected by patient DNA (which can be in significant excess over that of the microorganisms in wound samples). They cannot distinguish between viable and non-viable pathogens and require expensive equipment that is still unsuited to point-of-care (POC) use. Additionally, sequence databases are often biased towards pathogenic organisms, thus resulting in a significant underestimation of the true species diversity within a wound [57,58]. As such, their uptake into practice has been limited and they are used less in monitoring or routine screening, but rather as an investigative tool in clinically obvious acute infection [59]. Thus, the role of PCR is currently confirmatory rather than predictive. If, in the future, POC molecular techniques to detect infection become available, then this confirmatory role may become increasingly useful. However, further technological and automation improvements to reduce cost and time would be required to make this feasible or to use it in routine screens to detect incipient infection [60].

#### 2.3.4. Existing Biomarkers and Uses

An alternative, evolving approach to determine the presence of infection is the measurement of patient biomarkers of the immune system in response to incipient infection. Existing host-derived biomarkers of infection include C-Reactive Protein (CRP) [61], procalcitonin [62], hematologic markers [63], and more recently, the proposal to monitor lipocalin release from *N*-formyl-methionyl-leucyl-phenylalanine stimulated whole blood neutrophils [64]. All these markers are usually measured from blood or plasma samples and reflect systemic inflammatory status. Elevation associated with a local infection may suggest some systemic spread and the need for appropriate action (including intravenous antibiotic therapy). These markers are less useful in the early phases of local wound infections since the local markers that reach plasma are too dilute, and there has been no activation of significant systemic response, until the infection is, again, obvious at its source. Thus, local sampling of the wound itself is likely to yield sufficient biomass of the relevant host cells and cell products that otherwise would be highly diluted in blood samples. 

The discussion of blood versus wound sampling highlights a general issue in diagnostics, namely that of sensitivity and timing during the development of the target condition. The ideal in all diagnostic approaches is to detect changes as early as possible and this means both sensitivity to small amounts of marker and avoiding dilution or contamination in sampling. In the context of early detection of wound infection, local surface sampling is usually both convenient and non-dilutive. In contrast, systemic sampling appears more relevant for deep undrained surgical wounds for pragmatic reasons. The local biomarkers may not be the same as the systemic biomarkers, thus site and sampling should not be separated from the consideration of which biomarkers to evaluate. In this regard, wound infection diagnosis has a major advantage in that in most cases, the source of the sample is easily accessed.

#### 2.3.5. Electronic Noses and Imaging

The importance of anaerobes can lead to changes in volatile compounds emerging from the wound [65] and thus the potential for detection via electronic noses and similar technology. The advantage of such approaches is that they are non-invasive, potentially suitable for continuous monitoring and if sufficiently sensitive, potentially able to provide predictive data for incipient infections. Many such applications have been demonstrated using in vitro models [66] but the approach remains more difficult to apply in the clinical setting both in terms of sample acquisition and location of apparatus. These devices are generally not yet portable or suitable for point-of-care use. As the technology is driven by alternative uses, it is likely that improvements in sensors and portability will find their way to wound care applications in the coming decade. An alternative non-invasive approach is imaging, either thermal or ultraviolet. Multi-spectral analysis has the potential to track size, general biochemical markers, and fluorescent metabolites [67]. Imaging relies on powerful fluor- and chromophores produced by infecting organisms. These include porphyrins and pyocyanins, which can be distinguished from host autofluorescence. While fluorescence is capable of detecting a wide variety of porphyrin-producing wound bacteria (red fluorescence) and pyocyanin-producing *Pseudomonas aeruginosa* (cyan fluorescence), it is dependent on operator experience to distinguish the many sources of autofluorescence in wounds. This approach can be of significant benefit in locating bacterial “hotspots” in a wound to guide debridement and effective bioburden/biofilm removal, but it does not necessarily detect incipient infection. An extension of this approach is to stain the wound using materials that are specifically bound by biofilm components. Reports include the use of dyes used for plaque staining for teeth to stain biofilm in wounds. While elegant, these approaches serve a similar purpose as fluorescence techniques in detecting bioburden/biofilm to guide effective debridement, without facilitating the determination of infection status.

Such advances in microbial detection technologies and devices highlight the significance and progress that is being made in this field. In terms of ideal clinical requirements, related devices would be non-invasive and simple to use (by practitioners at all levels of expertise), would identify any potential foci of infection (including biofilm), would be sufficiently sensitive to detect incipient (early, non-obvious) infections, and would provide immediate outputs that guide a practitioner in providing optimal wound care such as effective local wound hygiene. While not all of these criteria are presently met, progress continues, and new approaches continue to evolve including a host-directed infection detection technology that is described in this paper.

### 2.4. Wound Healing: An Overview

Wound healing is a complex, highly regulated process comprising four definite phases: homeostasis, inflammation, proliferation, and maturation [68]. Acute, healthy wounds progress through all stages of wound healing with each phase properly activating the next. In contrast, chronic wounds do not progress normally but rather stagnate in the inflammatory phase [69]. The causes of entry into stasis are not understood, but contributing factors appear to include biofilm, proteolytic activity, and/or continuous re-injury via pressure, ischemia or other vascular deficits that reduce blood flow. These stasis events are often associated with continuous activation of inflammatory cells in the wound and so they can appear infected even if bioburden is low. Biofilm is now widely considered to be the main cause of persistent inflammation and delayed healing in chronic, hard-to-heal wounds [1,5].

#### 2.4.1. The Role of Inflammation in Wound Healing and Chronic Wounds

These observations suggest that a common aspect of wound stasis is sustained inflammation that persists because resolution is not initiated due to constant stimulus. Biofilm is now recognized as a constant stimulus, provoking a hyper-inflammatory state that prevents wound healing. Inflammation is an essential, innate immune response involving pathogen clearance as well as tissue breakdown and removal of cellular, extra-cellular and pathogenic debris. The inflammatory phase of wound healing involves a complex and overlapping cascade of molecular signals that ultimately facilitate leukocyte (monocyte and neutrophils) infiltration of the wound bed to mount a rapid and robust antimicrobial response [70]. During the inflammatory phase, platelet aggregation is followed by infiltration of leukocytes into the wound site, which are then found throughout the wound in varying degrees of vitality. Similarly, invading microorganisms can be found both within tissue, outside the confines of the wound bed, and in the wound dressing. Depending on the number and virulence of microorganisms encountered, the immune cells are either active and attracted to sites of infection, inactivated by pathogens, or are engulfing and lysing pathogens [71]. 

Once pathogens are cleared, immune cells orchestrate remodeling primarily through tissue degradation and formation through the activation of fibroblasts and endothelium. As such, an imbalance (excessive or reduced numbers) of inflammatory cells may have profound effects on downstream cell migration, proliferation, differentiation, and ultimately, the quality and duration of the overall healing response. Crucially, successful tissue repair requires the resolution of the inflammatory response for healing to progress to the proliferative stage [72,73]. The lack of resolution should be an indicator of persistent pro-inflammatory signaling or an imbalance in the regulation of immune cells at the site. Persistent organisms, biofilms or repeated injury can provide this pro-inflammatory stimulus, while the lysis of immune cells and the cleavage of signals and growth factors is one cause of the dysregulation of the cellular response to healing. A key source of the destructive inflammatory proteases is lysed neutrophils.

#### 2.4.2. The Role of Neutrophils in the Inflammatory Phase

Neutrophils are polymorphonuclear, phagocytic leukocytes that are part of the early host immune response against invading pathogens. They are recruited from peripheral blood initially, and later from bone from marrow in response to “find me” signals including damage-associated molecular patterns (DAMPs), hydrogen peroxide, lipid mediators, adenosine, and chemokines released from regions of injury or infection [74]. Neutrophils, like other myeloid cells, are not homogeneous and even more phenotypes are being recognized, which are related to tissue, age and phase of inflammation. 

**Figure 1:**
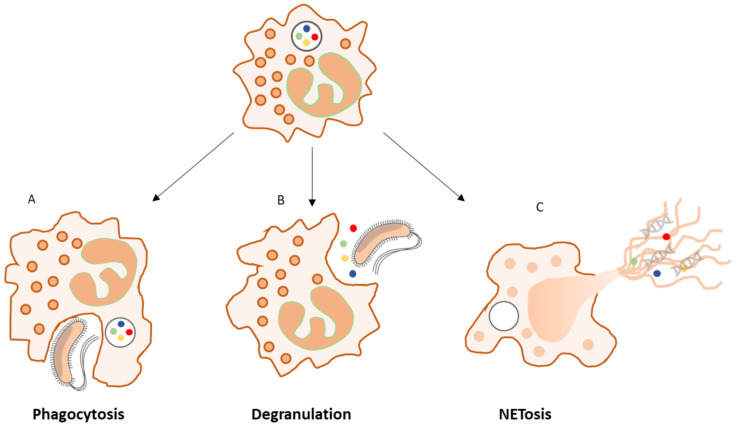
Intra- and extracellular neutrophil killing mechanisms. During phagocytosis (**A**), microorganisms are encapsulated within phagosomes. Pathogens are then killed by reactive oxygen species (ROS) or antimicrobial proteins. These antimicrobial proteins may be released from neutrophil granules into the extracellular milieu to kill pathogens by degranulation (**B**). Highly activated neutrophils can eliminate extracellular microorganisms by releasing neutrophil extracellular traps (NETs) (**C**). NETs are composed of core DNA to which histones, proteins, and enzymes (e.g., myeloperoxidase (MPO) and human neutrophil elastase (HNE)) are attached. Such structures immobilize pathogens near granules and facilitate phagocytosis of trapped microorganisms by other cells. NETosis and its role in biofilm-related infection are discussed in Section 2.5.3.

Neutrophils represent the most abundant inflammatory cells to infiltrate a wound in the early inflammatory phase of healing, where their primary function is to clear microorganisms to prevent infection and remove debris via a variety of mechanisms including phagocytosis, the release of toxic granules (degranulation), or the release of neutrophil extracellular traps (NETs) [68] (Figure 1). 

Whilst neutrophils play a crucial role in re-establishing tissue homeostasis via pathogen phagocytosis and macrophage recruitment, excessive neutrophil activity may lead to an overproduction of reactive oxygen species (ROS) and release of hydrolytic enzymes, causing extra cellular matrix (ECM) and cell membrane damage, ultimately resulting in premature cell senescence. The presence of ROS may also activate proteases (matrix metalloproteinases (MMPs) and serine proteases) and simultaneously inactivate protease inhibitors. Most of these effects are due to NETosis and neutrophil lysis, both of which release granules to the extracellular space. Both phenomena are associated with stimuli such as biofilm which are not susceptible to intracellular processing. The effect of granule release is to degrade ECM and growth factors which cause wounds to become chronic (or static) due to lack of structure, growth stimulus, and sustained immune activity [75].

Clearance of neutrophils begins with their apoptosis and subsequent engulfment by macrophages; a process known as efferocytosis [76]. This is critical because neutrophil contents are particularly potent in tissue degradation and their ordered destruction is important to homeostasis. Failure to activate neutrophil efferocytosis can lead to secondary necrosis where the neutrophils lyse, resulting in the release of pro-inflammatory cytotoxic molecules and proteases that increase tissue damage [70]. However, not all neutrophils are cleared by macrophages. Recent studies have shown that a subset of neutrophils leave the wound site through interstitial migration, or re-entry into the vasculature via the process of “reverse neutrophil migration” [77]. The purpose may be, amongst others, to transport captured pathogen cells to central immune organs such as the lymph nodes and the marginal zone of the spleen for antigen presentation [78]. 

Timely clearance of neutrophils is critical because it precedes resolution of inflammation. Neutrophil persistence, often itself is a response to microbial biofilm persistence, leads to a prolonged inflammatory state and thus non-healing wounds, due in part to the abundance of antimicrobial enzymes and peptides that degrade tissue and stall healing [70].

#### 2.4.3. Neutrophil Granules: A Rich Source of Proteases and Peroxidases 

Neutrophilic granules are located in the cytoplasm as small packages encapsulated by a lipid bilayer membrane. They contain multi-functional assemblages of proteins able to perform intracellular translocation, rapid alteration of neutrophil plasma membrane composition, extracellular discharge, cell–cell communication, and deployment of antimicrobial functionalities. Granules are classified based on the time at which they are formed during granulopoiesis, protein markers and dye affinity. Specifically, there are three types of neutrophil granule: (i) primary or azurophilic (markers include: myeloperoxidase (MPO), human neutrophil elastase (HNE), Cathepsin G (CatG), azurocidin); (ii) secondary or specific granules (lipocalin 2, lactoferrin); and (iii) tertiary or gelatinase granules (matrix metalloproteinase-9 (MMP-9), neutrophil collagenase) [79,80] (Table 1). Lysozyme is found in primary, secondary (co-located with lactoferrin [81]) and tertiary granules. The complement of proteases carried by neutrophils and other myeloid cells has multiple purposes. At one level it is to lyse pathogens, at another it is to allow these cells to pass through tissue or degrade intracellular proteins, or indeed other cells. The terms gelatinase, collagenase or indeed elastase are over-simplifications in that these enzymes are rarely truly specific and are almost always present in mixtures.

Neutrophils contain proteolytic enzymes (including serine proteases) which, along with MPO, define the primary granules. As pre-stored agents, neutrophil serine proteases can be quickly deployed in reaction to microbial challenge, to degrade internalized microbes, or upon release from activated neutrophils. Serine proteases are important contributors to the physiological response to infection, both as antimicrobial agents and as immunomodulators [82].

Uncontrolled HNE is known to be responsible for tissue loss and degeneration. Well-known examples include chronic lung diseases such as cystic fibrosis or chronic obstructive pulmonary disease. In wounds, proteolysis from host-derived enzymes fulfills a similar role in that it reverses or halts regenerative processes and degrades growth factors [83]. These factors further increase the total protease activity within the wound and exacerbate the host tissue damage. HNE thus impedes keratinocyte migration causing delayed healing [84]. 

### 2.5. Scenarios of Wound Healing

The inflammatory response following tissue injury or damage plays a crucial role in both normal and interrupted healing. Here, we examine three scenarios of wound healing. Firstly, the healing wound, where activation of the innate immune system results in the successful resolution of the inflammatory phase of healing and the wound progresses to remodeling. The second and third scenarios examine the status and impact of the wound when the innate immune system is overwhelmed by pathogens, under conditions of both early (acute) and prolonged (chronic) local infection.

#### 2.5.1. Scenario 1: The Healing Wound

**Figure 2:**
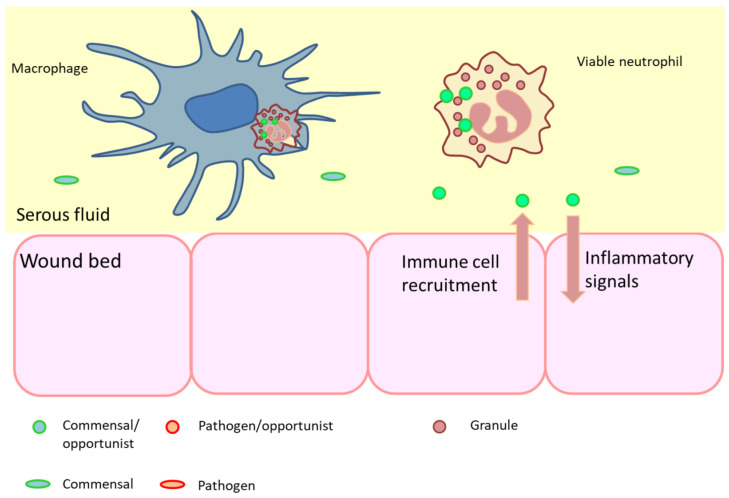
In a normally healing wound, microbial contamination will invariably occur, but good blood supply, immune cell function and nutrition will, in most cases, prevent progression to infection. When an adequate immune function is present, organisms that stimulate immune response are cleared by viable neutrophils through phagosomal lysis, after which the neutrophil itself undergoes apoptosis and is cleared by macrophages (preventing leakage of cell materials). Signaling that attracts immune cells is related to tissue injury, secretion of metabolites and microbial surface patterns. Since commensals generally cause no harm (e.g., invade tissue), immune recruitment is limited.

The innate immune system is activated immediately following injury or tissue damage, setting in motion a local inflammatory response that includes the recruitment of inflammatory cells from the circulation. Neutrophils promptly accumulate at the site of tissue injury, where their principal role is to phagocytose pathogens [80]. During physiological wound repair, neutrophils undergo apoptosis after completion of their various functions and are then subject to local macrophage uptake to trigger the transition out of the inflammatory phase (Figure 2).

**Figure 3:**
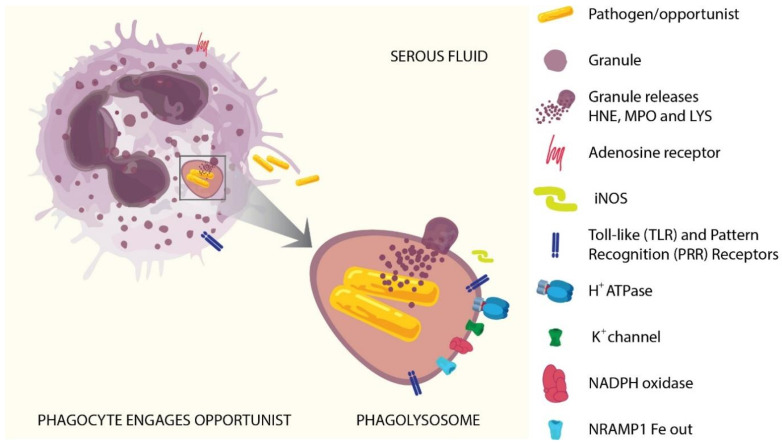
Neutrophil response to a potential pathogen (e.g., Enterobacteriaceae, or *Pseudomonas aeruginosa*). Upon contact with a pathogen, engulfment at the cell surface results in formation of a pre-lysosome to which function is added by fusion with various granules. Cation inward transport is activated, supplying K^+^ to exchange lytic enzymes occupying heparin sites on granules, and to exchange for H^+^ at later stages for maturation. At the membrane interface, transport is energized by H^+^-ATPase, which promotes pH reduction. In parallel, granules fuse and release lytic enzymes in concentrated form at the pathogen surface where they can cause local pore formation (resulting in loss of pH control). Up-regulation of NADPH oxidase supports oxidative burst via H_2_O_2_ production. Cl^−^ ion influxed with K^+^ is oxidized to hypochlorite (HOCl). While oxidative burst is considered to be an antibacterial process, chlorination is also a means of inactivating human antimicrobial enzymes [86,89], thus detoxifying the lysosome once the pathogen is killed. Abbreviations: HNE = human neutrophil elastase; MPO = myeloperoxidase; LYS = lysozyme; iNOS = inducible nitric oxide synthase; ATPase = H^+^ pump driven by ATP; NADPH = reduced (hydrogen form) of nicotinamide adenine dinucleotide phosphate; NRAMP1 Fe out = natural resistance-associated macrophage protein 1.

When a neutrophil encounters a microorganism, phagocytosis stimulates the maturation of the phagolysosome. Digestive antimicrobial enzymes which are held in granules are then recruited to the phagolysosome and their contents are transferred to it via fusion. These enzymes have exposed amines on their surfaces and are normally held in granules in an inactive form via electrostatic interactions with the anionic sulfated proteoglycan granule matrix (heparin-like) core of the granule [80]. Upon release into the phagolysosome, the presence of hypertonic potassium ions (K^+^) allows the release and activation of the enzymes. Other control measures such as elastase inhibitory peptides are also removed [85,86]. The action of the granular enzymes at the bacterial surface is accentuated by the phagosomal membrane conforming tightly to the bacterial surface, forcing granule contraction, which potentiates local pore-forming action. Subsequent acidification acts via the pore to ensure the loss of bacterial cytoplasmic pH control, elevating pH to a level optimal for neutral proteases, which are also activated by K^+^ driven into the vacuole to compensate the charge across the membrane [87,88]. Figure 3 illustrates the lysosome activation process.

In a successful interaction, the neutrophil with its dead bacterial contents becomes apoptotic and is cleared by a macrophage [75]. Digestive enzymes in the macrophage inactivate neutrophil contents, and most importantly, their lytic enzymes. In processes that are still poorly understood, the immune system is able to select the degree of digestion such that either the antigens are partially preserved and presented to the adaptive immune system (e.g., via dendritic cells), or all contents are maximally digested via the necrotic pathway with minimal antigen preservation [90]. 

#### 2.5.2. Scenario 2: Acute (Early Onset) Infection

**Figure 4:**
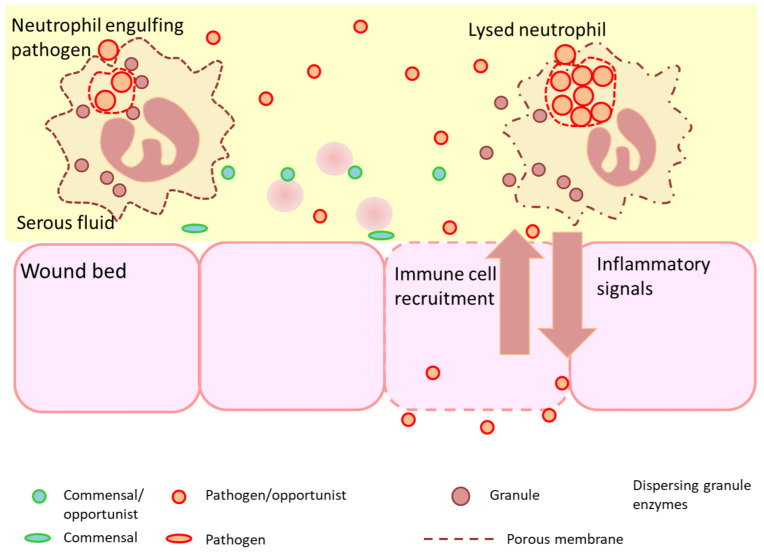
Early onset acute infection is associated with the lysis of immune cells by excess numbers of microorganisms, or the presence of organisms secreting toxins or virulence (pathogenicity) factors that inhibit immune function or injure host cells in the wound bed (e.g., pore-forming toxins). The polymicrobial nature of complex wounds will vary and may be dominated by fewer species over time if selective conditions arise (e.g., hypoxia, intra-microbial competition, use of antibacterial drugs). Toxins can also affect the wound bed and injure tissue. The potency and concentration of toxins are one of the factors that dictate the number of organisms necessary to establish an infection [92].

Pathogens may defeat neutrophils at various stages, either by permeabilizing membranes to prevent the formation of gradients or pH change, or by interrupting granule recruitment or maturation [44]. Mechanisms of evasion are many and have been widely studied in models involving *Mycobacterium tuberculosis*, *Klebsiella pneumoniae* and *Staphylococcus aureus*. Figure 4 illustrates the conditions under which neutrophils are unable to contain the microorganisms present. In this scenario, opportunistic commensals or pathogens are metabolically active in the planktonic form and multiply as saprophytes initially in wound debris before actively invading the wound bed. If perfusion to the wound bed is inadequate, neutrophil recruitment at the site will be limited. When these neutrophils encounter microorganisms and engulf them, the microorganisms are not efficiently killed, as they disable the phagosome and continue multiplication, drawing nutrients from the neutrophil. As microbial numbers increase, the phagosome is breached and the neutrophil lysed, releasing both microbial and neutrophil cell contents into the wound milieu [91].

#### 2.5.3. Scenario 3: Chronic (Prolonged, Local) Infection

The major determinant of the onset and outcome of microbial infection is the ability of the infecting organisms to overcome host innate defenses. This is dictated by the number of organisms, their virulence expression, their protection in biofilm communities or their ability to disable/evade immune response [1,16,93,94,95]. Hypoxia, devitalized tissue, biofilm, microbial toxins, viral co-infection, cancer, cancer treatments, obesity, diabetes, or foreign matter can weaken local innate immune cells and hamper the killing of phagocytosed organisms. Similarly, impairment of immune response through inadequate blood supply, or immune suppression reduces the ratio of neutrophils and other immune cells to pathogens and thus the probability of clearance [96]. When pathogens gain advantage either through abundance, pathogenicity or host weakness, immune cells become ineffective. This is particularly true where biofilm dominates within a wound environment, provoking a hyper-inflammatory state where neutrophil toxins and enzymes are unable to inactivate bacteria within the biofilm, and instead destroy host tissue and provide additional nutrition for mature biofilm [5]

The presence of biofilm poses a very different challenge to immune cells [21]. The main aspect of this is the size of the microbial community and its essential insolubility due to its matrix of extracellular polymeric substances (EPS). Biofilm communities may be many times larger than immune cells which means that there is no way for neutrophils to engulf the biofilm-protected microorganisms. Phagocytosis or engulfment works well for planktonic or isolated bacteria (i.e., once released from biofilm) that are typically one-tenth or less the diameter of the immune cell. Where the target is approximately the size of a mammalian cell, adhesion and cell–cell pore formation is used to kill the cell, followed by injection of digestive enzymes such as granzyme (e.g., natural killer cells and t-cells use this mechanism with tumor cells). In contrast, biofilm communities are larger and resemble a macro-parasite, yet the response of the immune system is similar in terms of physical cell disposition. Namely, attraction to the surface and the release of granules with lytic enzymes at the surface [97].

**Figure 5:**
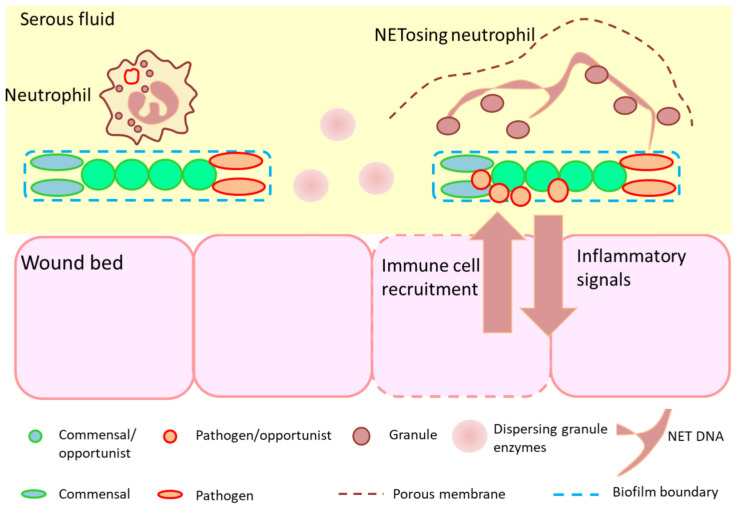
Biofilm stimulates a hyper-inflammatory immune response, leading to lysis, leakage and release of granular enzymes that are destructive to host tissue. Biofilm either develops at the wound site or may originate from biofilm fragments shed from other sources [104]. Neutrophils respond to biofilm in a manner analogous to parasite responses, namely, to lyse at the interface and form NETs, in which the neutrophil DNA is spread over the object surface to distribute granules and place them at the interface. The granules then release their enzymes onto the surface where they can potentially degrade the matrix. In most biofilms, this is ineffective and tends to only release proteases to the surrounding tissue and add DNA to the biofilm agglomeration where it is often used by the biofilm organisms themselves as EPS. This general scheme illustrates how both lysis of immune cells and NETosis lead to the release of host enzymes in the extracellular space [98,99,100,101,102,103,105].

This is apparent in the neutrophil NETosis response (Figure 5). Neutrophil extracellular traps are structures that become apparent where the well-known engulfment processes do not function [97,98,99]. These appear to be a coordinated set of processes, resulting in the neutrophil lysing in such a way that its DNA strands form a large network that distributes the lytic granules over a wider area [100]. Local microorganisms can be caught in these strands and the granules brought into contact with the microbial surface. NET formation is an aspect of hyper-inflammation (also referred to as “frustrated phagocytosis”) and represents a form of last resort response to an evasive pathogen [101].

In the context of biofilm, the NETosis response likely reflects the fact that the biofilm structure is too large to be engulfed yet persists in emitting stimulatory signals leading to both neutrophil attraction and the NETosis response [102]. This is perpetuated, in that the biofilm structures and organisms within are tolerant to the enzymes released by neutrophils, thus successive waves of neutrophils are lost in this way. Furthermore, the DNA released by neutrophils is often incorporated into the biofilm and is not degraded by the DNAse that neutrophils also release. Thus, the NETosis response is often ineffective and may also help build biofilm via the incorporation of the resulting debris into biofilm EPS [100,103]. 

Both the biofilm response and the lytic response to planktonic or isolated pathogens are associated with the release of neutrophil contents [106]. With increasing infection, more neutrophils are attracted, thus more are lysed or NETosed. Thus, there is a positive correlation between infection progression and the number of lysed neutrophils [107].

Wound progression towards macroscopically detectable infection is characterized by initial phases in which microorganism numbers are low. Either the outgrowth of opportunists, or the presence of organisms with virulence factors, initiates tissue injury and neutrophil activation. Should these immune cells fail to contain this initial insult, cell lysis begins along with tissue injury and stimulus. This subsequently attracts more immune cells, and an “incipient infection” condition is present. Ultimately, if the immune response fails, tissue injury, excess dead immune cells and microorganisms combine to form pus and other exudates, which are macroscopically recognizable as an infection (where current practice leads to intervention) [44]. 

Given the extent of processes that take place prior to the production of visible pus, it becomes reasonable to propose that infection leading to pus formation represents an extreme degree of infection and not just a first sign of “infection”. Indeed, in most other areas of medicine, waiting for visible signs would be unacceptable. Just as it is possible to measure blood pressure or blood sugar, our understanding of wound biology and immunology is now such that we can observe most of the steps that lead to the failure of local immune surveillance before there are extreme numbers of microorganisms present. It is then a reasonable proposition to ask if this knowledge can be used to monitor wounds and detect deleterious transitions while they are still free of visible signs of infection. 

## 3. Towards a New Approach to Wound Infection Detection

Given the known limitations of diagnosing infection through clinical judgment, microbial culture, molecular techniques, and the relative lack of sensitivity of blood biomarkers, alternative approaches to early detection of infection in wounds could have significant clinical utility [11]. To detect early-stage infections, a new approach is needed that is compatible with current wound monitoring techniques, and independent of wound status or colonizing/infecting organisms. A host-directed approach fulfills these requirements, owing to both the constancy of host biomarkers and sample supply in the form of wound exudate. Here, we outline progress in the use of immunological markers to both predict and confirm the onset of infection in wounds. 

### 3.1. Neutrophil Enzymes as Markers to Detect Wound Infection

In previous sections, we have described how the immune system responds quickly to the presence of pathogens that are capable of harming host tissue. This response is proportional to pathogen presence and increases dramatically if the pathogens are either numerous or able to kill or lyse the immune cells initially present. If pathogens are successful, then an infection will only be sustained while the infiltrating neutrophils are continuously lysed leading to the loss of their internal enzymes to the wound fluid.

Neutrophil enzymes have, therefore, the potential to be used as markers of infection [108]. A number of clinical pilot studies have demonstrated increases in activity of the neutrophil enzymes MPO [109], HNE [110], CatG [110,111] in wound fluid samples from infected wounds, as defined by clinical judgment and/or microbiological analyses. Given that clinical judgment backed by culture is the current gold standard, these studies established that these neutrophil markers were elevated in “obvious” infections. However, there were also elevated enzymes in some samples that were not yet “considered to be infected”. These observations prompted the question as to whether these samples were derived from “incipient infections” that were not yet visible.

Collectively, these studies suggested that using multiple neutrophil (immune) markers lead to a combined parameter that was highly associated with the infected wound state both in terms of specificity and sensitivity. That each marker was not in a fixed proportion to the others is a matter of immunological interest and may suggest that neutrophils adapt their compliment of enzymes to the nature of the pathogen. Alternatively, it may indicate that they differ in stage of infection or in their stability in the samples taken. Nonetheless, the sum of these markers was more robust than individual markers in a subsequent clinical study [9]. Investigating the reasons for this observation remains the focus of longer-term studies.

In the process of evaluating these parameters, it became apparent that how the sample was taken, the nature of fluids, and timing relative to other wound treatments were also important. Having taken all factors into account, these studies, nonetheless, highlighted the issue that the gold-standard, microbiology, or clinical opinion may not be reliable. In general, “false positives” outnumbered “false negatives”. A number of samples classified as “false positive” using biomarkers may indeed be suggesting that “infection” is more common than we currently accept or that the result was simply observed at an earlier stage of infection development. In current practice, cryptic infection evades detection for some time and as noted earlier, microbiology remains difficult to interpret, especially in the context of biofilm. Thus, these studies suggested to us that there may be a degree of under-diagnosis or early, slowly developing infection that is frustrating in current wound care practice.

To detect these markers many technologies are available such as ELISA, lateral flow antibody systems and more advanced proteomics. However, while these approaches may be necessary for proteins such as cytokines or growth factors, neutrophil enzymes can signal their own presence through their inherent activity. Selective substrates for these functions can be optimized to demonstrate the activity of the enzymes. The issue is more generally to identify a detection principle and a format—soluble or in situ—and to define competing or inhibitory effects from samples or sampling systems. When these aspects are optimized, the addition of appropriate indicator reagents allows the detection of changes in the enzyme activities of MPO, HNE and lysozyme (LYS) via color, fluorescence emission, or substrate loss. 

Detecting enzyme activity is inherently more cost-effective than the physical methods described above. Detection can be visible; the materials are inexpensive, and the sampling (via swab) is the same as that currently performed for microbiological investigation. Thus, it may enable the monitoring of wounds using simple techniques [11]. The clinical utility of such chromogenic reagents was demonstrated in a study [9] in which samples from 81 patients were assessed. Post hoc data analysis revealed 3 patterns of enzymatic response associated with clear infection: at least one highly elevated enzyme, two moderately elevated (HNE, LYS) or at least one high (MPO) and one moderate (HNE or LYS). 

If such chromogenic technology outlined above is designed to be instant or rapid in response to host biomarkers, and readily observable to the naked eye of an HCP, then this opens up the possibility of POC testing for wound infection assessment.

### 3.2. A Window of Opportunity 

**Figure 6:**
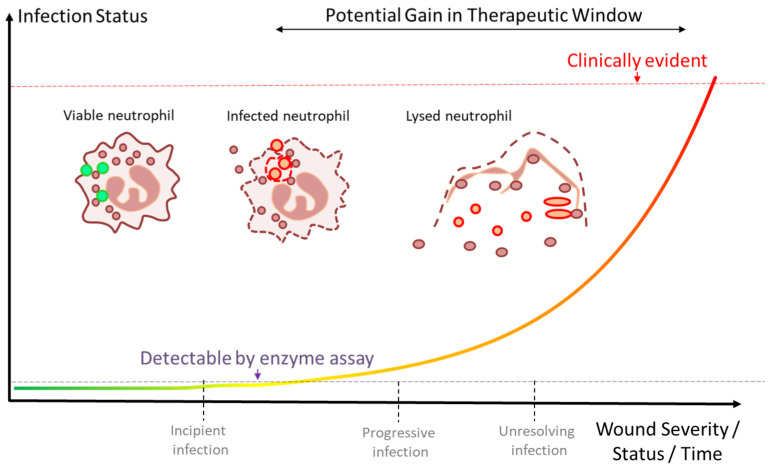
Time course of wound infection. On the X-axis, the stage of infection, as determined from visible signs, is shown with progressively worsening infection severity to the right. The quantity and activity of detectable markers are shown on the Y-axis, with more markers being released as infection status worsens and more immune cells are recruited. Two levels are indicated—the level at which infection is visible to the eye through edema, redness and suppuration (red dotted line) and the level at which these markers can be detected by appropriate assays and potentially, POC tests (purple dotted line).

The degree to which these markers indicate incipient or progressive infection provides a window of warning in advance of the infection progressing, i.e., to the point where it is visually obvious (Figure 6). This window is an opportunity in which measures can be introduced to reduce bioburden and provide better conditions for a more optimized host response. As microbial multiplication can be exponential, early measures may have a substantial impact on the size of microbial populations [2].

### 3.3. The Four Pillars of Wound Infection Detection

There are four general opportunities for improved wound infection detection in wound management:

I. The first can be considered as “**screening**”. In this setting, inexpensive reagents are used regularly to ascertain infection status. If, over time, higher levels are detected, an incipient infection may be suspected, and appropriate action taken. Such an inflection point in biomarker levels is also a reasonable point to initiate any other measures such as additional wound hygiene [112], antisepsis and antimicrobial dressings. Monitoring via screening is particularly relevant to fast-changing settings such as surgical wounds, where early intervention could happen on the scale of hours or days. In the post-surgical setting, regular testing of fluids either from drains or sutures may prove prudent as a means to initiate therapy whilst bacterial numbers are low, if a wound does not immediately progress to healing.
II. The second opportunity is more relevant to longer-term or hard-to-heal wounds and can be considered “**providing more certainty**” or “**disambiguation**” of an unclear clinical picture. This applies more to situations where wound healing is delayed, but classical signs of infection are not apparent. Under these conditions, sub-clinical levels of infection may be interfering with the resolution and healing processes. However, because these are not visible, they may go untreated. This may be particularly the case for biofilm which may be underestimated by microbiological analysis. In such cases, the use of biomarkers as measures of infection may detect the underlying cause of wound stasis and provide a new impetus or therapy direction [2,113]. Indeed, the fact that biofilm induces a “frustrated” hyper-inflammatory state, detection of elevated enzymes in chronic wounds could indirectly confirm the presence of biofilm.
III. The third opportunity is “**monitoring following diagnosis**”. In this setting, the impact or success of the measures taken should be assessed in real time if possible. Thus, ineffective wound hygiene measures can be recognized by a resurgence in biomarkers before a return to suppuration is observed. In various studies, the application of antimicrobial agents has been associated with a reduction in biomarkers, suggesting that the use of these substances reduced lysis of immune cells. Alternatively, no change or a further increase in biomarkers would signal a failure of therapy and possibly resistance to the agent(s) in use.
IV. The fourth opportunity relates to the common problem of persister cells, biofilm, and the re-emergence of infection after cessation of therapy. Thus, “**monitoring of resolved infections**”, especially in unstable patients is a means to validate remission or to detect progression or reversion to infection before it is severe. This is particularly relevant to those with immune suppression or multiple wounds.

**Figure 7:**
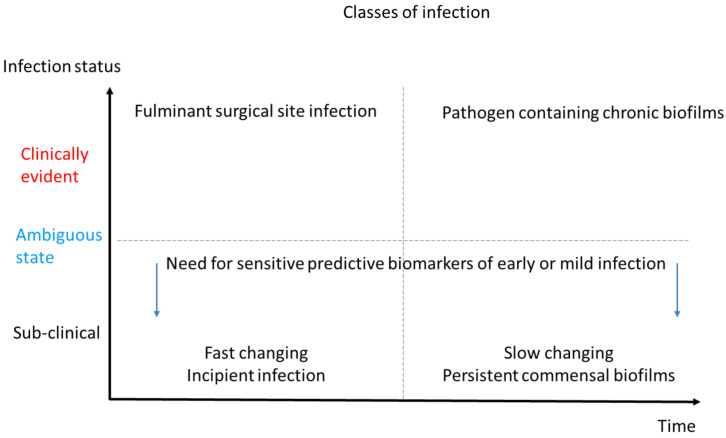
Classification of infection in terms of time, duration, and severity. The X-axis indicates two rates of infection progression—early onset, and prolonged—in terms of the time required for the signs to become visible. The Y-axis indicates three broad conditions of infection from sub-clinical (not visible) to ambiguous (some signs but not obvious) and clear advanced infection. The diagnostic challenge is to detect infections in the sub-clinical state or when wound state appears ambiguous. Should it be possible to detect incipient infection or sub-clinical persistent biofilms, this will have a major impact on treatment paradigms.

These concepts are illustrated in Figure 7 in which infection scenarios are illustrated in terms of quadrants relating the rate of infection development to the apparent clinical status. The use of biomarkers is particularly useful to detect infections in the lower quadrants, i.e., in the early phases of an acute post-surgical infection, or to disambiguate a chronic infection (Pillars I and II). The top quadrants of Figure 7 represent clinically obvious infections that need to be treated; in this setting, relating to monitoring the success of therapy allows for adequate application of therapy (Pillar III) and hygiene to prevent relapse and exposure to high microbial numbers (Pillar IV). Thus, in the therapeutic setting, waiting for the return of purulence to conclude that a measure is ineffective represents a loss of time and unnecessary tissue injury. Simple, colorimetric biomarker monitoring allows this conclusion to be reached much sooner and helps justify the application of more effective measures while the microbial burden remains low.

## 4. Summary

Focusing on the degree of immune activation within a wound enables a clearer indication of wound infection status, since this reflects the degree to which the organisms present are damaging the wound bed irrespective of their types or numbers. The concept of infection is transformed from arbitrary to distinct, by looking at when the host immune response is overwhelmed on a personalized level. This approach to wound infection classification is sympathetic to the innate differences encountered in host immune response between individuals [4,44,68,69,94,114].

Neutrophil granular enzymes such as MPO, HNE, LYS and CatG are tools of the first responder neutrophil cells to local infection [68,86,96,107]. Clinical studies have demonstrated that the presence of multiple neutrophil enzymes is indicative of infection, that an increase in enzyme levels over the baseline corresponds to infection onset [106], and that these enzymes decrease following antimicrobial treatment indicating resolution of infection [114,115]. In contrast to systemic blood markers such as CRP and procalcitonin, neutrophil enzymes are present locally and sampling from wound fluid is non-invasive [9,106,116]. Changes in CRP levels generally indicate systemic spread and are associated with other clinically apparent signs of infection [63], whilst neutrophil enzymes elevate locally well before systemic signs elevate [9]. Detecting the early onset of infection via host-responsive neutrophil enzymes provides a means to monitor a wound, initiating appropriate therapy, perhaps by topical antisepsis, on early indication of emerging infection, rather than waiting for conventional signs such as odor, inflammation, pain, or suppuration. These are evident in an established infection for which antibiotic therapy may be indicated. 

Thus, passive monitoring of neutrophil enzymes as detectors of incipient infection, using a rapid POC test, may be a means to provide an immediate response to an emerging problem, not an established one. This is in contrast to conventional microbiology and remote analysis where results arrive several days later. Given the exponential nature of microbial growth, this time gained is potentially critical and could lead to a reduction in microbial burden and a re-invigoration of host responses before tissue damage occurs. The application of this paradigm should lead to more prompt, appropriate, and cost-effective local wound care (wound hygiene), reduce antibiotic usage, and hence reduce the opportunity for the development of antibiotic resistance in wound care.

Approaching wound infection in this way offers significant potential to improve interventions and outcomes. We think it is important for our profession to adopt our suggestion of considering visually apparent infection as a severe manifestation and to begin to see infection as a process that is part of a quantifiable spectrum from early-to-late, or initial-to-severe. This change in approach could be of major benefit to patients debilitated by hard-to-heal, chronic wounds. 

## Figures and Tables

**Table 1:** Abundant human granule proteins [79,80].

Protein Name	Protein Function
**Primary granules–Main marker: Myeloperoxidase (MPO)**	
Myeloperoxidase (MPO)	Hypochlorite deactivation of microbial and granular proteins
Human neutrophil elastase (HNE)	Serine protease, immune cell activation, C5a- reactions
Cathepsin G (Cat G)	Antibacterial Serine protease, complement C3 cleavage
Azurocidin	Antibacterial activity, chemoattractant
Neutrophil defensins	Antimicrobial activities
Myeloblastin	Serine protease supporting neutrophil migration
Lysozyme	Lysis of bacterial cell walls, also detected in primary granules of progenitor cells [79]
CD63 antigen	Surface receptor of TIMP1
Cap57 (BPI)	Bactericidal protein
**Secondary (specific) granules–Main marker: Lactoferrin**	
Lactoferrin	Iron binding and transport
Lipocalin 2	Iron-trafficking, involvement in innate immunity and apoptosis
Lysozyme	Lysis of bacterial cell walls
Chitinase-3-like protein 1	Carbohydrate(chitin)-binding lectin
Cytochrome B558	Membrane component of the phagocyte O_2_-producing NADPH oxidase
Collagenase	Cellular migration
CD11b/CD18	Adhesion complex (Integrin), endocytosis of R-G-D-C3b bound particles
fMLP-R	Formyl peptide receptor 1
**Tertiary granules–Main marker: Gelatinase**	
Matrix metalloproteinase (MMP)-9	Supports migration by cleaving collagen/gelatin
MMP-8	Cleavage of collagens
Lysozyme	Lysis of bacterial peptidoglycan
Cathelicidin	Antibacterial pro-peptide
Ficolin-1	PAMP receptor
**Quaternary granules/Secretory Vesicles**	
fMLP-R	Formyl peptide receptor 1
CD11b/CD18	Adhesion complex (Integrin), endocytosis of R-G-D-C3b bound particles
Cytochrome B558	Membrane component of the phagocyte O_2_-producing NADPH oxidase
Alkaline phosphatase	Detoxification of lipopolysaccharide (LPS), anti-inflammatory
CR1	Complement receptor type 1 (CR1), mediates binding to particles that activated complement

## Data Availability

Not applicable.

## References

[1] Wolcott R.D., Rhoads D.D., Dowd S.E. (2008). Biofilms and chronic wound inflammation. J. Wound Care.

[2] Bowler P.G., Duerden B.I., Armstrong D.G. (2001). Wound microbiology and associated approaches to wound management. Clin. Microbiol. Rev..

[3] Thomson P.D., Smith D.J. (1994). What is infection?. Am. J. Surg..

[4] Morton L.M., Phillips T.J. (2016). Wound healing and treating wounds: Differential diagnosis and evaluation of chronic wounds. J. Am. Acad. Dermatol..

[5] Hurlow J., Bowler P.G. (2022). Acute and chronic wound infections: Microbiological, immunological, clinical, and therapeutic distinctions. J. Wound Care.

[6] Haalboom M., Blokhuis-Arkes M.H.E., Beuk R.J., Meerwaldt R., Klont R., Schijffelen M.J., Bowler P.G., Burnet M., Sigl E., van der Palen J.A.M. (2019). Culture results from wound biopsy versus wound swab: Does it matter for the assessment of wound infection?. Clin. Microbiol. Infect..

[7] Bowler P.G. (2003). The 10(5) bacterial growth guideline: Reassessing its clinical relevance in wound healing. Ostomy Wound Manag..

[8] Cutting K.F., White R. (2004). Defined and refined: Criteria for identifying wound infection revisited. Br. J. Community Nurs..

[9] Blokhuis-Arkes M.H.E., Haalboom M., van der Palen J., Heinzle A., Sigl E., Guebitz G., Beuk R. (2015). Rapid enzyme analysis as a diagnostic tool for wound infection: Comparison between clinical judgment, microbiological analysis, and enzyme analysis. Wound Repair Regen..

[10] Schiffer D., Verient V., Luschnig D., Blokhuis-Arkes M.H.E., van der Palen J., Gamerith C., Burnet M., Sigl E., Heinzle A., Guebitz G.M. (2015). Lysozyme-responsive polymer systems for detection of infection. Eng. Life Sci..

[11] Schiffer D., Tegl G., Heinzle A., Sigl E., Metcalf D., Bowler P., Burnet M., Guebitz G.M. (2015). Enzyme-responsive polymers for microbial infection detection. Expert Rev. Mol. Diagn..

[12] Tegl G., Schiffer D., Sigl E., Heinzle A., Guebitz G.M. (2015). Biomarkers for infection: Enzymes, microbes, and metabolites. Appl. Microbiol. Biotechnol..

[13] Schiffer D., Tegl G., Vielnascher R., Weber H., Herrero-Rollett A., Sigl E., Heinzle A., Guebitz G.M. (2016). Myeloperoxidase-responsive materials for infection detection based on immobilized aminomethoxyphenol. Biotechnol. Bioeng..

[14] Metcalf D.G., Haalboom M., Bowler P.G., Gamerith C., Sigl E., Heinzle A., Burnet M.W.M. (2019). Elevated wound fluid pH correlates with increased risk of wound infection. Wound Med..

[15] Webb R. (2017). A chronic case of confusion. J. Wound Care.

[16] Edwards R., Harding K.G. (2004). Bacteria and wound healing. Curr. Opin. Infect. Dis..

[17] White R.J., Cutting K.F. (2006). Critical colonization--the concept under scrutiny. Ostomy Wound Manag..

[18] Bowler P.G., Davies B.J. (1999). The microbiology of infected and noninfected leg ulcers. Int. J. Dermatol..

[19] White R.J., Cutting K.F. (2008). Critical colonisation of chronic wounds: Microbial mechanisms. Wounds UK.

[20] Malone M., Bjarnsholt T., McBain A.J., James G.A., Stoodley P., Leaper D., Tachi M., Schultz G., Swanson T., Wolcott R.D. (2017). The prevalence of biofilms in chronic wounds: A systematic review and meta-analysis of published data. J. Wound Care.

[21] Wolcott R.D. (2017). Biofilms cause chronic infections. J. Wound Care.

[22] Bowler P.G. (2018). Antibiotic resistance and biofilm tolerance: A combined threat in the treatment of chronic infections. J. Wound Care.

[23] Chen L., Wen Y.M. (2011). The role of bacterial biofilm in persistent infections and control strategies. Int. J. Oral Sci..

[24] Stewart P.S., Costerton J.W. (2001). Antibiotic resistance of bacteria in biofilms. Lancet.

[25] Bowler P., Murphy C., Wolcott R. (2020). Biofilm exacerbates antibiotic resistance: Is this a current oversight in antimicrobial stewardship?. Antimicrob. Resist. Infect. Control.

[26] Vuong C., Kocianova S., Voyich J.M., Yao Y., Fischer E.R., DeLeo F.R., Otto M. (2004). A crucial role for exopolysaccharide modification in bacterial biofilm formation, immune evasion, and virulence. J. Biol. Chem..

[27] Costerton J.W., Stewart P.S., Greenberg E.P. (1999). Bacterial biofilms: A common cause of persistent infections. Science.

[28] Percival S.L., Hill K.E., Malic S., Thomas D.W., Williams D.W. (2011). Antimicrobial tolerance and the significance of persister cells in recalcitrant chronic wound biofilms. Wound Repair Regen..

[29] Wood T.K., Knabel S.J., Kwan B.W. (2013). Bacterial persister cell formation and dormancy. Appl. Environ. Microbiol..

[30] Bjarnsholt T. (2013). The role of bacterial biofilms in chronic infections. APMIS.

[31] Percival S.L., Bowler P.G. (2004). Biofilms and Their Potential Role in Wound Healing. Wounds.

[32] Bowler P. (2005). The role of bacterial communities in wound healing. J. Tissue Viability.

[33] Metcalf D., Bowler P., Parsons D., Dhanasekaran D., Thajuddi N. (2016). Wound Biofilm and Therapeutic Strategies. Microbial Biofilms.

[34] Attinger C., Wolcott R. (2012). Clinically Addressing Biofilm in Chronic Wounds. Adv. Wound Care.

[35] Høiby N., Ciofu O., Johansen H.K., Song Z.J., Moser C., Jensen P.Ø., Molin S., Givskov M., Tolker-Nielsen T., Bjarnsholt T. (2011). The clinical impact of bacterial biofilms. Int. J. Oral Sci..

[36] Geisinger E., Isberg R.R. (2017). Interplay Between Antibiotic Resistance and Virulence During Disease Promoted by Multidrug-Resistant Bacteria. J. Infect. Dis..

[37] Glaudemans A.W., Uçkay I., Lipsky B.A. (2015). Challenges in diagnosing infection in the diabetic foot. Diabet. Med..

[38] Li S., Renick P., Senkowsky J., Nair A., Tang L. (2021). Diagnostics for Wound Infections. Adv. Wound Care.

[39] Bertesteanu S., Triaridis S., Stankovic M., Lazar V., Chifiriuc M.C., Vlad M., Grigore R. (2014). Polymicrobial wound infections: Pathophysiology and current therapeutic approaches. Int. J. Pharm..

[40] Wu Y.-K., Cheng N.-C., Cheng C.-M. (2019). Biofilms in Chronic Wounds: Pathogenesis and Diagnosis. Trends Biotech..

[41] Bamberg R., Sullivan P., Conner-Kerr T. (2002). Diagnosis of wound infections: Current culturing practices of U.S. Wound care professionals. Wounds.

[42] Rondas A.A., Halfens R.J., Schols J.M., Thiesen K.P., Trienekens T.A., Stobberingh E.E. (2015). Is a wound swab for microbiological analysis supportive in the clinical assessment of infection of a chronic wound?. Future Microbiol..

[43] Fazli M., Bjarnsholt T., Kirketerp-Møller K., Jørgensen B., Andersen A.S., Krogfelt K.A., Givskov M., Tolker-Nielsen T. (2009). Nonrandom distribution of Pseudomonas aeruginosa and Staphylococcus aureus in chronic wounds. J. Clin. Microbiol..

[44] MacLeod A.S., Mansbridge J.N. (2016). The Innate Immune System in Acute and Chronic Wounds. Adv. Wound Care.

[45] Enoch S., Grey J.E., Harding K.G. (2006). ABC of wound healing. Non-surgical and drug treatments. Br. Med. J. Clin. Res. Ed..

[46] Bowler P., Davies B.J. (1999). The Microbiology of Acute and Chronic Wounds. Wounds.

[47] Malone M., Johani K., Jensen S.O., Gosbell I.B., Dickson H.G., Hu H., Vickery K. (2017). Next Generation DNA Sequencing of Tissues from Infected Diabetic Foot Ulcers. EBioMedicine.

[48] Maugeri G., Lychko I., Sobral R., Roque A.C.A. (2019). Identification and Antibiotic-Susceptibility Profiling of Infectious Bacterial Agents: A Review of Current and Future Trends. Biotech. J..

[49] Tong S.Y.C., Davis J.S., Eichenberger E., Holland T.L., Fowler V.G. (2015). Staphylococcus aureus infections: Epidemiology, pathophysiology, clinical manifestations, and management. Clin. Microbiol. Rev..

[50] Gupta D., Agarwal R., Aggarwal A.N., Singh N., Mishra N., Khilnani G.C., Samaria J.K., Gaur S.N., Jindal S.K., Pneumonia Guidelines Working Group (2012). Guidelines for diagnosis and management of community- and hospital-acquired pneumonia in adults: Joint ICS/NCCP(I) recommendations. Lung India.

[51] Allaband C., McDonald D., Vázquez-Baeza Y., Minich J.J., Tripathi A., Brenner D.A., Loomba R., Smarr L., Sandborn W.J., Schnabl B. (2019). Microbiome 101: Studying, Analyzing, and Interpreting Gut Microbiome Data for Clinicians. Clin. Gastroenterol. Hepatol..

[52] De Oliveira F.P., Pires B.M.F.B., de Cássia Ferreira de Almeida Silva K., de Carvalho B.T.F., Teixeira L.A., de Paula G.R., de Oliveira B.G.R.B. (2017). Prevalence, Antimicrobial Susceptibility, and Clonal Diversity of Pseudomonas aeruginosa in Chronic Wounds. J. Wound Ostomy Cont. Nurs..

[53] Wolcott R.D., Dowd S.E. (2008). A rapid molecular method for characterising bacterial bioburden in chronic wounds. J. Wound Care.

[54] Verbanic S., Shen Y., Lee J., Deacon J.M., Chen I.A. (2020). Microbial predictors of healing and short-term effect of debridement on the microbiome of chronic wounds. npj Biofilms Microbiomes.

[55] Hu H., Jacombs A., Vickery K., Merten S.L., Pennington D.G., Deva A.K. (2015). Chronic Biofilm Infection in Breast Implants Is Associated with an Increased T-Cell Lymphocytic Infiltrate: Implications for Breast Implant–Associated Lymphoma. Plast. Reconstr. Surg..

[56] Kommedal Ø., Lekang K., Langeland N., Wiker H.G. (2011). Characterisation of polymicrobial clinical samples using a set of group-specific briad-range primers targeting the 16rRNA gene followed by DNA sequencing and RipSeq analysis. J Med Micro..

[57] Clarridge J.E. (2004). Impact of 16S rRNA gene sequence analysis for identification of bacteria on clinical microbiology and infectious diseases. Clin. Microbiol. Rev..

[58] Percival S.L., Hill K.E., Williams D.W., Hooper S.J., Thomas D.W., Costerton J.W. (2012). A review of the scientific evidence for biofilms in wounds. Wound Repair Regen..

[59] Yang S., Rothman R.E. (2004). PCR-based diagnostics for infectious diseases: Uses, limitations, and future applications in acute-care settings. Lancet Infect. Dis..

[60] Petralia S., Conoci S. (2017). PCR Technologies for Point of Care Testing: Progress and Perspectives. ACS Sens..

[61] Sproston N.R., Ashworth J.J. (2018). Role of C-Reactive Protein at Sites of Inflammation and Infection. Front. Immunol..

[62] Faix J.D. (2013). Biomarkers of sepsis. Crit. Rev. Clin. Lab. Sci..

[63] Biron B.M., Ayala A., Lomas-Neira J.L. (2015). Biomarkers for Sepsis: What Is and What Might Be?. Biomark. Insights.

[64] Venge P. (2018). Human neutrophil lipocalin (HNL) as a biomarker of acute infections. Upsala J. Med. Sci..

[65] Slade E.A., Thorn R.M., Young A.E., Reynolds D.M. (2022). Real-time detection of volatile metabolites enabling species-level discrimination of bacterial biofilms associated with wound infection. J. Appl. Microbiol..

[66] Saviauk T., Kiiski J.P., Nieminen M.K., Tamminen N.N., Roine A.N., Kumpulainen P.S., Hokkinen L.J., Karjalainen M.T., Vuento R.E., Aittoniemi J.J. (2018). Electronic nose in the detection of wound infection bacteria from bacterial cultures: A proof-of-principle study. Eur. Surg. Res..

[67] Janowska A., Davini G., Romanelli M., Oranges T., Iannone M., Dini V. (2021). The association between pH and fluorescence as non-invasive diagnostic tools in chronic wounds. Int. J. Lower Extrem. Wounds.

[68] Ellis S., Lin E.J., Tartar D. (2018). Immunology of Wound Healing. Curr. Dermatol. Rep..

[69] Schultz G.S., Chin G.A., Moldawer L., Diegelmann R.F., Fitridge R., Thompson M. (2011). Principles of Wound Healing. Mechanisms of Vascular Disease: A Reference Book for Vascular Specialists.

[70] Rodrigues M., Kosaric N., Bonham C.A., Gurtner G.C. (2018). Wound Healing: A Cellular Perspective. Physiol. Rev..

[71] Eming S.A., Krieg T., Davidson J.M. (2007). Inflammation in Wound Repair: Molecular and Cellular Mechanisms. J. Investig. Dermatol..

[72] Landén N.X., Li D., Ståhle M. (2016). Transition from inflammation to proliferation: A critical step during wound healing. Cell. Mol. Life Sci..

[73] Su Y., Richmond A. (2015). Chemokine Regulation of Neutrophil Infiltration of Skin Wounds. Adv. Wound Care.

[74] Larouche J., Sheoran S., Maruyama K., Martino M.M. (2018). Immune Regulation of Skin Wound Healing: Mechanisms and Novel Therapeutic Targets. Adv. Wound Care.

[75] Bratton D.L., Henson P.M. (2011). Neutrophil clearance: When the party is over, clean-up begins. Trends Immunol..

[76] De Oliveira S., Rosowski E.E., Huttenlocher A. (2016). Neutrophil migration in infection and wound repair: Going forward in reverse. Nature Rev. Immunol..

[77] Rosales C. (2018). Neutrophil: A Cell with Many Roles in Inflammation or Several Cell Types?. Front. Physiol..

[78] Sheshachalam A., Srivastava N., Mitchell T., Lacy P., Eitzen G. (2014). Granule Protein Processing and Regulated Secretion in Neutrophils. Front. Immunol..

[79] Cassatella M.A., Östberg N.K., Tamassia N., Soehnlein O. (2019). Biological Roles of Neutrophil-Derived Granule Proteins and Cytokines. Trends Immunol..

[80] Cramer E.M., Breton-Gorius J. (1987). Ultrastructural localization of lysozyme in human neutrophils by immunogold. J. Leukoc. Biol..

[81] Majewski P., Majchrzak-Gorecka M., Grygier B., Skrzeczynska-Moncznik J., Osiecka O., Cichy J. (2016). Inhibitors of Serine Proteases in Regulating the Production and Function of Neutrophil Extracellular Traps. Front. Immunol..

[82] McCarty S.M., Percival S.L. (2013). Proteases and Delayed Wound Healing. Adv. Wound Care.

[83] Buchstein N., Hoffmann D., Smola H., Lang S., Paulsson M., Niemann C., Krieg T., Eming S.A. (2009). Alternative proteolytic processing of hepatocyte growth factor during wound repair. Am. J. Pathol..

[84] Uribe-Querol E., Rosales C. (2017). Control of Phagocytosis by Microbial Pathogens. Front. Immunol..

[85] Segal A.W. (2005). How neutrophils kill microbes. Ann. Rev. Immunol..

[86] Lukacs G.L., Rotstein O.D., Grinstein S. (1990). Phagosomal acidification is mediated by a vacuolar-type H(+)-ATPase in murine macrophages. J. Biol. Chem..

[87] Nordenfelt P., Tapper H. (2011). Phagosome dynamics during phagocytosis by neutrophils. J. Leukoc. Biol..

[88] Nguyen G.T., Green E.R., Mecsas J. (2017). Neutrophils to the ROScue: Mechanisms of NADPH Oxidase Activation and Bacterial Resistance. Front. Cell. Infect. Microbiol..

[89] Vyas J.M., Van der Veen A.G., Ploegh H.L. (2008). The known unknowns of antigen processing and presentation. Nature Rev. Immunol..

[90] Kobayashi S.D., Malachowa N., DeLeo F.R. (2017). Influence of Microbes on Neutrophil Life and Death. Front. Cell. Infect. Microbiol..

[91] Wolcott R., Costerton J.W., Raoult D., Cutler S.J. (2013). The polymicrobial nature of biofilm infection. Clin. Microbiol. Infect..

[92] Bowler P.G. (2002). Wound pathophysiology, infection and therapeutic options. Ann. Med..

[93] Wolcott R., Sanford N., Gabrilska R., Oates J.L., Wilkinson J.E., Rumbaugh K.P. (2016). Microbiota is a primary cause of pathogenesis of chronic wounds. J. Wound Care.

[94] Hornef M.W., Wick M.J., Rhen M., Normark S. (2002). Bacterial strategies for overcoming host innate and adaptive immune responses. Nat. Immunol..

[95] Wilgus T.A., Roy S., McDaniel J.C. (2013). Neutrophils and Wound Repair: Positive Actions and Negative Reactions. Adv. Wound Care.

[96] Branzk N., Lubojemska A., Hardison S.E., Wang Q., Gutierrez M.G., Brown G.D., Papayannopoulos V. (2014). Neutrophils sense microbe size and selectively release neutrophil extracellular traps in response to large pathogens. Nat. Immunol..

[97] Yipp B.G., Petri B., Salina D., Jenne C.N., Scott B.N.V., Zbytnuik L.D., Pittman K., Asaduzzaman M., Wu K., Meijndert H.C. (2012). Infection-induced NETosis is a dynamic process involving neutrophil multitasking in vivo. Nat. Med..

[98] Hahn S., Giaglis S., Chowdury C.S., Hösli I., Hasler P. (2013). Modulation of neutrophil NETosis: Interplay between infectious agents and underlying host physiology. Sem. Immunopathol..

[99] Wong S.L., Demers M., Martinod K., Gallant M., Wang Y., Goldfine A.B., Kahn C.R., Wagner D.D. (2015). Diabetes primes neutrophils to undergo NETosis, which impairs wound healing. Nat. Med..

[100] Manfredi A.A., Ramirez G.A., Rovere-Querini P., Maugeri N. (2018). The Neutrophil’s Choice: Phagocytose vs Make Neutrophil Extracellular Traps. Front. Immunol..

[101] Van Avondt K., Hartl D. (2018). Mechanisms and disease relevance of neutrophil extracellular trap formation. Eur. J. Clin. Investig..

[102] Radic M. (2014). Clearance of Apoptotic Bodies, NETs, and Biofilm DNA: Implications for Autoimmunity. Front. Immunol..

[103] Parsons D., Metcalf D.G. (2014). Understanding local barriers to wound healing. Next-Generation Antimicrobial Dressings: AQUACEL™ Ag+ Extra™ and Ribbon.

[104] De Bont C.M., Boelens W.C., Pruijn G.J.M. (2019). NETosis, complement, and coagulation: A triangular relationship. Cell. Mol. Immunol..

[105] Brinkmann V., Reichard U., Goosmann C., Fauler B., Uhlemann Y., Weiss D.S., Weinrauch Y., Zychlinsky A. (2004). Neutrophil Extracellular Traps Kill Bacteria. Science.

[106] Wang J. (2018). Neutrophils in tissue injury and repair. Cell Tissue Res..

[107] Németh T., Sperandio M., Mócsai A. (2020). Neutrophils as emerging therapeutic targets. Nat. Rev. Drug Discov..

[108] Hasmann A., Wehrschuetz-Sigl E., Marold A., Wiesbauer H., Schoeftner R., Gewessler U., Kandelbauer A., Schiffer D., Schneider K.P., Binder B. (2013). Analysis of myeloperoxidase activity in wound fluids as a marker of infection. Ann. Clin. Biochem..

[109] Hasmann A., Gewessler U., Hulla E., Schneider K.P., Binder B., Francesko A., Tzanov T., Schintler M., van der Palen J., Guebitz G.M. (2011). Sensor materials for the detection of human neutrophil elastase and cathepsin G activity in wound fluid. Exp. Dermatol..

[110] Hasmann A., Wehrschuetz-Sigl E., Kanzler G., Gewessler U., Hulla E., Schneider K.P., Binder B., Schintler M., Guebitz G.M. (2011). Novel peptidoglycan-based diagnostic devices for detection of wound infection. Diagn. Microbiol. Infect. Dis..

[111] Murphy C., Atkin L., Swanson T., Tachi M., Tan Y.K., Vega de Ceniga M., Weir D., Wolcott R. (2020). International consensus document. Defying hard-to-heal wounds with an early antibiofilm intervention strategy: Wound hygiene. J. Wound Care.

[112] Metcalf D.G., Bowler P.G. (2016). Clinician perceptions of wound biofilm. Int. Wound J..

[113] Witko-Sarsat V., Rieu P., Descamps-Latscha B., Lesavre P., Halbwachs-Mecarelli L. (2000). Neutrophils: Molecules, Functions and Pathophysiological Aspects. Lab. Investig..

[114] Thakur A., Mikkelsen H., Jungersen G. (2019). Intracellular Pathogens: Host Immunity and Microbial Persistence Strategies. J. Immunol. Res..

[115] Kennedy A.D., DeLeo F.R. (2009). Neutrophil apoptosis and the resolution of infection. Immunol. Res..

[116] Pedersen F., Marwitz S., Holz O., Kirsten A., Bahmer T., Waschki B., Magnussen H., Rabe K.F., Goldmann T., Uddin M. (2015). Neutrophil extracellular trap formation and extracellular DNA in sputum of stable COPD patients. Respir. Med..

